# Decomposing the effects of ocean environments on predator–prey body-size relationships in food webs

**DOI:** 10.1098/rsos.180707

**Published:** 2018-07-18

**Authors:** Tomoya Dobashi, Midori Iida, Kazuhiro Takemoto

**Affiliations:** 1 Department of Bioscience and Bioinformatics, Kyushu Institute of Technology, Iizuka, Fukuoka 820-8502, Japan; 2 Center for Marine Environmental Studies (CMES), Ehime University, Bunkyo-cho 2-5, Matsuyama 790-8577, Japan

**Keywords:** food web, predator–prey body size ratio, ocean environment, climate change

## Abstract

Body-size relationships between predators and their prey are important in ecological studies because they reflect the structure and function of food webs. Inspired by studies on the impact of global warming on food webs, the effects of temperature on body-size relationships have been widely investigated; however, the impact of environmental factors on body-size relationships has not been fully evaluated because climate warming affects various ocean environments. Thus, here, we comprehensively investigated the effects of ocean environments and predator–prey body-size relationships by integrating a large-scale dataset of predator–prey body-size relationships in marine food webs with global oceanographic data. We showed that various oceanographic parameters influence prey size selection. In particular, oxygen concentration, primary production and salinity, in addition to temperature, significantly alter body-size relationships. Furthermore, we demonstrated that variability (seasonality) of ocean environments significantly affects body-size relationships. The effects of ocean environments on body-size relationships were generally remarkable for small body sizes, but were also significant for large body sizes and were relatively weak for intermediate body sizes, in the cases of temperature seasonality, oxygen concentration and salinity variability. These findings break down the complex effects of ocean environments on body-size relationships, advancing our understanding of how ocean environments influence the structure and functioning of food webs.

## 1. Introduction

The structure of food webs, which indicate who eats whom, has attracted much attention in ecology because they are important for understanding the functioning and stability of ecosystems against environmental perturbations (e.g. climate change) in the context of both basic scientific research (e.g. structure–stability relationships [1,2]) and applied ecology (e.g. biodiversity maintenance and environmental assessment [3,4]). However, quantifying the effect of body size (or mass) on food-web structure is challenging [5,6]. Body size influences predator–prey interactions; moreover, it is expected to be an important factor in characterizing the physiological parameters (e.g. metabolic rate (oxygen consumption, energy demand) [7,8], lifespan [9,10] and animal space use [11,12]) that determine food web dynamics. In this context, the body-size relationships between predators and their prey are of particular interest [13–15] because they affect the strength of interactions [16,17], feeding rate [18], trophic level [19,20] and food web dynamics [17,21].

The relationship of body size with environmental factors is also important [22,23]. For example, the temperature–size rule [24] states that body size shrinks with increasing temperature; however, this rule is subject to debate [25]. Previous studies suggest that environmental factors (e.g. changing temperature) alter predator–prey body-size relationships [15]. In fact, several studies have reported the association between environmental factors and predator–prey body-size relationships. For instance, Lurgi *et al*. [26] showed that climate warming significantly reduces predator–prey mass ratios (i.e. the magnitude by which predators are larger than prey) at low and intermediate elevations in mountain ecosystems. Moreover, using a large-scale dataset of marine food webs [27], the effect of temperature on predator–prey body-size relationships was investigated at a global scale. Although Barnes *et al*. [28] reported that body-size relationship was not significantly influenced by temperature, Gibert & DeLong [29] demonstrated that temperature alters body-size relationships by controlling for the hierarchical structure of the data in statistical analyses.

However, more focused investigations are required to reach a conclusion on the association between environmental factors and predator–prey body-size relationships. Previous studies have primarily focused on the effects of current temperature and warming temperature. However, other environmental factors also need to be considered because the relationship between such factors and temperature are expected from previous studies. This issue demonstrates the need for controlling the potentially confounding effects of temperature on body-size relationships. For example, ocean warming might also lead to a decline in dissolved O_2_ in the ocean interior (ocean deoxygenation) [30]. The impact of climate change differs between the surface and deeper layers of the ocean [31]. Ocean warming intensifies the global water cycle, causing ocean salinity to increase [32]. The global phytoplankton population (primary production) has declined over the past century, with this long-term declining trend being associated with increasing sea surface temperatures [33].

These oceanographic parameters are also expected to influence predator–prey body-size relationships. For example, the body size of fish varies with depth [34]. Salinity might increase body size because larger fish are subjected to less osmotic stress than smaller fish [35]. A decline in the chlorophyll concentration might affect population dynamics and body size because it reflects a decrease in food availability or primary production [36]. In addition, a decline in the body size of marine fish might be explained by the hypothesis that the oxygen (energy) demand for maintaining body size is not being met, because ocean deoxygenation results from climate warming [37]; however, this suggestion is subject to debate [38,39].

Energy demand (i.e. metabolic rate) is a key factor explaining changes in body size. For instance, several studies [40,41] have indicated that optimal body size is selected based on the balance between resource supply and energy demand. Various environmental factors influence metabolic rate. Temperature accelerates the metabolic rate [42], even though an increase in metabolic rate due to temperature might be saturated [43,44]. Salinity also has large metabolic costs for fishes [45]. Thus, body size is expected to alter according to variation in the metabolic rate in response to these environmental changes.

The variability and seasonality of oceanographic parameters might affect body size. Ecological interaction networks (predator–prey interactions, in this case) vary along environmental gradients, in addition to time and space [46]. A previous study [47] reported that body size increases with the seasonality of temperature in ectotherms, whereas another study [48] indicated that increasing seasonality in temperature reduces body size in arthropod species. Higher variability in resources (e.g. primary production) generally indicates that the supply of resources is unstable. In such cases, the overall supply declines; thus, body size is expected to vary.

The development of field observation technologies and the improvement of infrastructures for databases and data sharing have increased the availability of such oceanographic data. For example, large quantities of data have been collected and are available from the World Ocean Database [49].

In this study, we hypothesized that ocean environments affect predator–prey body-size relationships. Particularly inspired by previous studies [28,29], we re-evaluated how these different ocean environments influence body-size relationships. Using a large-scale dataset of predator–prey interactions in marine food webs and a database of oceanographic parameters, we comprehensively investigated how ocean environments contribute to predator–prey body-size relationships using random effects models.

## 2. Material and methods

### 2.1. Dataset

Following a previous study [29], data on predator–prey body-size relationships in marine food webs [27] were downloaded from the EcoData Retriever database [50] on 4 August 2016. The units of all the predator and prey masses were converted to grams (g). Moreover, we also extracted the depth (m), habitat type (e.g. coastal bay and open ocean), references, primary production (PP_mean_), variability (standard deviation) of primary production (PP_var_), and latitude and longitude at each observation site. PP_mean_ and PP_var_ were estimated from the surface concentration of chlorophyll *a* pigment [27] (see also esapubs.org/archive/ecol/E089/051/metadata.htm). According to the references, we obtained the original research papers, and manually extracted the observation periods (i.e. start date (month/year) and end date (month/year)). The observation periods of two of the 30 observation points (defined based on coordinates and depths) were not known; thus, the data on predator–prey interactions at these observation points were excluded.

Based on these observation parameters (i.e. latitude and longitude, depth and observation periods), we obtained the following oceanographic parameters at a spatial resolution of 1° grid from the World Ocean Atlas 2013 (v. 2) (www.nodc.noaa.gov/OC5/woa13/): annual mean temperature (*T*_mean_) (°C), temperature variability (standard deviation of temperature; *T*_var_), annual mean (dissolved) oxygen concentration (O_2mean_) (ml l ^−l^), oxygen variability (standard deviation of oxygen concentration; O_2var_), annual mean salinity (*S*_mean_) (unitless) and salinity variability (standard deviation of salinity; *S*_var_). We followed a set of designated procedures. In this database, the data on *T*_mean_, *T*_var_, *S*_mean_ and *S*_var_ were available for each decadal period (e.g. 1985–1994 and 1995–2004). According to the observation periods, we selected the most suitable decadal period, and downloaded the annual data for the period. For example, we used the data for the decadal period of 1995–2004 for the geographical location *Apalachicola Bay Florida* (electronic supplementary material, table S1) because the observation period was between 1999 and 2002. However, we considered multiple decadal periods for the locations *Atlantic Ocean* and *Europe Celtic Sea ecosystem* because the observation periods were long term. In particular, we obtained the annual data for 1965–1974, 1975–1984 and 1985–1994 for the location *Atlantic Ocean* (observation period of 1973–1990), and obtained the average values over these data. The annual data for 1975–1984 and 1985–1994 were used for the location *Europe Celtic Sea ecosystem* (observation period of 1977–1994). For O_2mean_ and O_2var_, the decadal periods were not available; thus, we downloaded the single annual datasets for O_2mean_ and O_2var_, respectively. Based on the coordinates (latitudes and longitudes) and depths, we extracted these oceanographic parameters at the observation points from the downloaded datasets. However, the parameters have not always been available at the depths of the observation points (at deep sea, in particular). Thus, we selected the nearest depth level at which the oceanographic parameters were available. We then calculated the difference between the actual depths (i.e. the depths described in the original dataset [27]) and depths at which the parameters were extracted. Finally, we integrated these data and parameters (see electronic supplementary material, table S1) and investigated 33 511 predator–prey body-size relationships.

### 2.2. Data analyses

Following a previous study [29], we used linear mixed-effect models to evaluate the contribution of each factor affecting prey mass, while controlling for the hierarchical structure of the data. The analyses were performed in R software (v. 3.4.3; www.R-project.org) using packages *lme4* (v. 1.1.15) and *lmerTest* (v. 2.0.36). For all analyses, the masses of predators and preys were log-transformed. The habitat types and predator identity (species) were considered as random intercepts and random slopes, respectively. This approach was used because of the variability in predator–prey mass ratio due to animal types and habitats [14,20], and to control for the error associated with the allometric estimates of predator mass [29] (i.e. the slope or power law exponent of the curve for prey mass versus predator mass). The random effect for predator identity was also used to account for differences in sampling effort among species [51]. The quantitative variables were normalized to the same scale, with a mean of 0 and standard deviation of 1, using the *scale* function in R before the analyses.

We constructed the full model, which was the model encompassing all explanatory variables (i.e. predator mass, depth, O_2mean_, O_2var_, PP_mean_, PP_var_, *S*_mean_, *S*_var_, *T*_mean_ and *T*_var_). In the full model, following a previous study [29], we also considered the interactive effects of log-transformed predator mass and each oceanographic parameter on log-transformed prey mass to evaluate the contribution of oceanographic parameters to the slope (i.e. power law exponent) of the curve for prey mass versus predator mass. The best model was selected using the sample-size-corrected version of the Akaike information criterion (AICc), using the package *MuMIn* (v. 1.40.4) in R. To avoid model selection bias, we adopted a model-averaging approach [52,53] using R packages *MuMIn* and *snow* (v. 0.4.2). We obtained the averaged model in the top 95% confidence set of models. The contribution (i.e. non-zero estimate) of each explanatory variable to log-transform prey mass was considered significant when the associated *p*-value was less than 0.05.

## 3. Results

We present the results of the full, best and averaged models (table 1). Predator body size was strongly associated with prey body size. Moreover, we found that the wide-ranging oceanographic environments affected predator–prey body-size relationships. In particular, the models suggested that depth, oxygen variability (O_2var_), primary production (PP_mean_) and temperature (*T*_mean_) were negatively associated with prey size, whereas the variability of primary production (PP_var_) was positively associated with prey size. These results suggest that prey size varies with these oceanographic parameters. The effect of salinity (*S*_mean_) was limited.

**Table 1.** Influence of explanatory variables on the prey body mass. The results of the full model, best model and averaged model are shown. *X*_mean_ and *X*_var_ indicate the mean and variability (standard deviation) of oceanographic parameter *X*, respectively. The abbreviations of oceanographic parameters are as follows: O_2_ (oxygen concentration), PP (primary production), *S* (salinity) and *T* (temperature). Prey mass and predator mass data were log-transformed. *R*^2^ is the conditional coefficient of determination for the mixed-effect models. s.e. is the standard error.

	full model			best model			averaged model		
variables	estimate	s.e.	*p*-value	estimate	s.e.	*p*-value	estimate	s.e.	*p*-value
predator mass	0.79	0.07	<2.2 × 10^–16^	0.77	0.06	<2.2 × 10^–16^	0.78	0.07	<2.2 × 10^–16^
predator mass × depth	0.36	0.09	2.3 × 10^–4^	0.35	0.08	4.7 × 10^–5^	0.37	0.09	7.6 × 10^–5^
predator mass × O_2mean_	0.14	0.05	8.8 × 10^–3^	0.13	0.05	5.2 × 10^–3^	0.14	0.06	0.012
predator mass × O_2var_	–0.02	0.05	0.76				0.03	0.05	0.51
predator mass × PP_mean_	0.30	0.05	3.0 × 10^–7^	0.33	0.05	8.0 × 10^–10^	0.31	0.06	3.0 × 10^–7^
predator mass × PP_var_	–0.10	0.04	0.015	–0.12	0.04	1.7 × 10^–3^	–0.12	0.04	4.3 × 10^–3^
predator mass × *S*_mean_	–0.08	0.05	0.12				–0.08	0.04	0.068
predator mass × *S*_var_	–0.24	0.03	<2.2 × 10^–16^	–0.21	0.02	<2.2 × 10^–16^	–0.22	0.03	<2.2 × 10^–16^
predator mass × *T*_mean_	0.19	0.05	7.5 × 10^–4^	0.13	0.04	8.4 × 10^–4^	0.15	0.06	9.5 × 10^–3^
predator mass × *T*_var_	0.08	0.03	0.012	0.10	0.03	9.5 × 10^–4^	0.10	0.04	5.6 × 10^–3^
depth	–1.10	0.29	1.8 × 10^–3^	–1.16	0.29	8.1 × 10^–3^	–1.23	0.32	1.5 × 10^–4^
O_2mean_	–0.11	0.08	0.17	–0.08	0.07	0.28	–0.11	0.10	0.30
O_2var_	–0.18	0.09	0.042	–0.21	0.08	0.014	–0.19	0.08	0.023
PP_mean_	–0.55	0.08	1.9 × 10^–8^	–0.60	0.07	2.6 × 10^–13^	–0.55	0.09	<2.2 × 10^–16^
PP_var_	0.44	0.13	2.4 × 10^–3^	0.46	0.10	2.3 × 10^–5^	0.40	0.12	1.0 × 10^–3^
*S*_mean_	0.05	0.11	0.69				0.04	0.12	0.74
*S*_var_	0.00	0.03	0.93	0.00	0.02	0.68	0.00	0.03	0.96
*T*_mean_	–0.44	0.09	6.0 × 10^–5^	–0.42	0.09	7.0 × 10^–5^	–0.38	0.10	2.0 × 10^–4^
*T*_var_	0.07	0.05	0.22	0.05	0.06	0.33	0.04	0.07	0.56
*R*^2^	0.95			0.96					
AICc	30 716			30 713					

**Figure 1.**
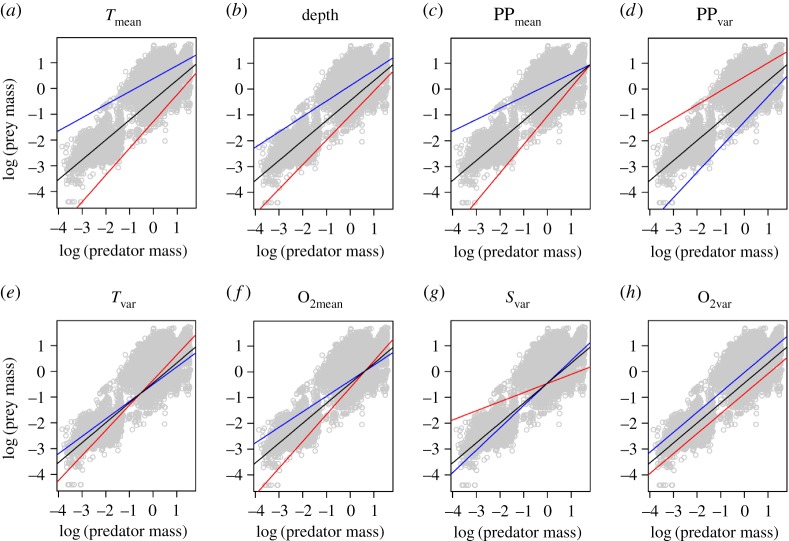
Effects of ocean environments on the relationship between predator mass and prey mass. Prey mass and predator mass data were log-transformed and normalized to the same scale, with a mean of 0 and standard deviation of 1, respectively. The symbols (grey-coloured open circles) correspond to the observed data. The red, black and blue lines are the predicted curves obtained from the best model at high, medium and low values of an oceanographic parameter, respectively. When obtaining the predicted curves from the best model, the variables other than an arbitrary parameter were fixed in the model, using their mean values in the dataset. (*a*) The effect of annual mean temperature (*T*_mean_). The high, medium and low values are 12.9, 6.0 and –0.9, respectively. (*b*) The effect of depth. The high, medium and low values are 2511, 1622 and 733, respectively. (*c*) The effect of mean primary production (PP_mean_). The high, medium and low values are 998.5, 702.3 and 406.1, respectively. (*d*) The effect of the variability (standard deviation) of primary production (PP_var_). The high, medium and low values are 136.6, 68.0 and 7.0, respectively. (*e*) The effect of the variability of temperature (*T*_var_). The high, medium and low values are 3.5, 0.9 and 0.0, respectively. (*f*) The effect of mean oxygen concentration (O_2mean_). The high, medium and low values are 7.3, 5.1 and 3.0, respectively. (*g*) The effect of the variability of salinity (*S*_var_). The high, medium and low values are 0.33, 0.06 and 0.00, respectively. (*h*) The effect of the variability of oxygen concentration (O_2var_). The high, medium and low values are 0.8, 0.3 and 0.0, respectively. The medium values correspond to the mean values in the dataset. The high and low values were generally selected based on the *Z*-values in the dataset; in particular, the *Z*-values for the high and low values indicate 2 and –2, respectively. However, there are some exceptions because of the standard deviations and minimum value in the dataset. In particular, the *Z*-values of 1 and –1 were considered for the high and low values of PP_mean_, respectively. The *Z*-values of 0.5 and –0.5 were used for the high and low values of depth, respectively. The minimum values (i.e. 0) in the dataset were considered for the low values of *T*_var_, PP_var_ and *S*_var_.

The interactive effects of predator size and oceanographic parameters indicated that wide-ranging oceanographic parameters affected the curve for prey size versus predator size. The full, best and averaged models suggested that depth, oxygen concentration (O_2mean_), PP_mean_, PP_var_, salinity variability (*S*_var_), *T*_mean_ and temperature variability (*T*_var_) determined the slope. In comparison, the interactive effects of oxygen variability and salinity were limited (table 1). Specifically, a relatively steeper slope was observed at greater depth (deeper sites), oxygen concentration, temperature and temperature variability. In comparison, a relatively shallower slope was observed for high variability of primary production. These results indicate that the effects of these oceanographic parameters on the body-size relationship differ with body size. High temperature, depth and primary production led to lower intercepts and steeper slopes (figure 1*a–c*). High variability of primary production led to a higher intercept and shallower slope (figure 1*d*). These results indicate that the effects of depth, primary production, variability of primary production and temperature on the body-size relationships were noticeable for small body sizes. More complex patterns were also observed. Oxygen concentration, salinity variability and temperature variability did not affect the intercepts (i.e. the effects of these parameters were limited at the mean of log-transformed predator size); however, they did affect the slopes. This result indicates that the effects of these oceanographic parameters were stronger for small and large body sizes and were relatively weak for intermediate sizes (at around the mean of log-transformed predator size). Moreover, these results indicate that the effect of these oceanographic parameters is inverted for intermediate body sizes. Temperature variability caused prey size to decrease when predator size was small; however, temperature variability caused prey size to increase when predator size was large (figure 1*e*). A similar pattern was also observed for oxygen concentration (figure 1*f*). Prey size increased with salinity variability when predators were small, but decreased with salinity variability when predators were large (figure 1*g*). Oxygen variability did not alter the slopes and only decreased intercepts (figure 1*h*).

## 4. Discussion

The results indicated that larger predators generally have larger prey, which is consistent with a number of previous studies (e.g. [13–15,20]). In contrast with a previous study [28], this study showed that the oceanographic parameters affected the predator–prey body-size relationship, although both studies were based on the same dataset [27]. This discrepancy might be mainly because the previous study only controlled for the effect of location and not for the hierarchical structure of the data in statistical analyses.

Similar to a previous study [29], we found that temperature altered the body-size relationship (figure 1*a*). Specifically, smaller predators tend to eat smaller prey items at higher temperatures. In comparison, the body size of prey was relatively unchanged for larger predators. The change in body size is probably explainable in the context of the temperature–size rule [24]. This correlation might also exist because the population of small-sized species (prey) increases with warming temperature [54]. However, the results presented here are not entirely consistent with the results of the previous study [29], which reported that relatively small predators eat larger prey at higher temperatures, while relatively larger predators eat smaller prey. This discrepancy might be due to differences in the datasets and data analyses between this study and previous study. Although both studies were based on the same dataset [27], we used temperature at around the depth of the sampling points, whereas the previous study simply used sea surface temperature. In addition, we investigated the effect of temperature on the body-size relationships, while controlling for the potentially confounding effects of the other oceanographic parameters.

Our results do not contradict the previous studies [28,29]. Rather, they provide complementary insights into the relationship between ocean environments and predator–prey body-size relationship. In particular, we showed that a variety of oceanographic parameters were associated with body-size relationship (table 1).

Prey body size was lower at deeper sites (figure 1*b*). This result is consistent with the fact that teleost body size decreases with depth because resource availability decreases with depth [34]. The effect of depth was more remarkable for smaller body sizes. This may be because the body surface area per body mass (the effect of water pressure) is larger for smaller organisms.

Smaller prey size was observed in areas with higher primary production, with this effect being significant for small body sizes (figure 1*c*). This result is consistent with the fact that the predator–prey mass ratio increases with increasing primary production because the population of small-sized species (primary consumers) increases due to high primary production [19]. The inverse effect was observed in the case of the variability of primary production (figure 1*d*). Higher variability in primary production indicates an unstable resource supply. Thus, the population of small-sized species might decrease remarkably forcing predators to eat relatively larger prey.

The effects of temperature variability, oxygen concentration and salinity variability were more complex. In particular, the effects of these parameters were inverted at around intermediate body sizes. These complex relationships were possibly observed because different mechanisms were mixed.

Temperature seasonality showed positive and negative effects on prey size for large and small predators, respectively (figure 1*e*). The positive effect might be because larger body sizes might be adapted to more seasonal environments because larger individuals have higher resistance to starvation in ectotherms [47]. In particular, energy stores increase with size faster than metabolic rate (i.e. fasting endurance hypothesis [55]). In addition, larger body size might be favoured in cold environments (i.e. Bergmann's rule [56]) because the surface–mass ratio is reduced (i.e. thermal inertia increases). On the other hand, the negative effect might result from the fact that relatively small aquatic species, including plankton, are adapted to meet higher metabolic demands with seasonal warming by reducing body size [48].

The reason why salinity seasonality affects the body-size relationship (figure 1*g*) is particularly unclear because few studies have been conducted on how salinity seasonality affects body size. Further examinations are required, although this might be related to the facts that large body size is favourable for increasing salinity tolerance (i.e. for decreasing osmotic stress) because the ratio of gill area to body weight decreases as the body weight of the fish increases [35] and salinity tolerance differs with respect to age and body size [45].

Prey size reduction due to a decrease in oxygen concentration for relatively large predators (figure 1*f*) might correspond to a decrease in marine fish size to meet the energy demand to maintain their body size in response to a decrease in oxygen concentration [37]. The increase in prey size due to a decrease in oxygen concentration for relatively small predators might be because ocean deoxygenation leads to a reduction in nutrient loads (i.e. starvation state) [57]. In particular, larger body sizes might be selected because larger individuals have higher resistance to starvation.

The increase in oxygen variability causing prey mass to decline (figure 1*h*) is also probably explained by a decrease in fish size due to ocean deoxygenation [37] because the availability of energy might decrease due to the unstable supply of oxygen (i.e. higher oxygen variability).

The variability in the body-size relationships according to ocean environments is related to changes in predation behaviour (and consequently, food web structure) due to environmental changes. In fact, several studies (e.g. [58,59]) have reported that differences in the ocean environment (e.g. oxygen) affect predator–prey interactions. Specifically, a shallower slope of the curve for prey mass versus predator mass suggests larger interaction strengths and fewer trophic levels in food webs [15,29]. Theoretical studies have suggested that larger interaction strengths [1,2] and fewer trophic levels [60] lead to a decline in ecosystem stability. Given the decrease in phytoplankton [33] and ocean deoxygenation [30] due to ocean warming, the results of the present study suggest that marine food webs are destabilized by climate change. However, more detailed examinations are required to make conclusions about the effects of ocean environments on predator–prey body-size relationships and ecosystem stability. Specifically, the analysis we present here has some limitations.

The oceanographic parameters used in the current study might be slightly different from actual observations because of the time and spatial resolution of the data. This issue exists because of the primary limitation of the World Ocean Atlas database. Moreover, we did not consider pH levels because no data on pH levels are available in the World Ocean Atlas database. Although pH levels are available in other databases (e.g. World Ocean Database; https://www.nodc.noaa.gov/OC5/WOD/pr_wod.html), the amount of data was not sufficient for data analyses (for calculating the statistical means and standard deviations, in particular). A number of studies have reported the relationship between ocean acidification (pH) and climate warming (e.g. [61]). Moreover, ocean acidification might alter predation behaviours [59,62]. Thus, future studies must consider pH levels when conducting more detailed examinations.

This study is limited in the context of predator–prey body-size relationships. To evaluate food web structure, it is important to consider food web topology. Ecological networks have been studied from a complex network perspective, inspired by the development of network science [63,64]. For example, several previous studies [52,53,65,66] have reported that current climate, climate change (global warming) and human activity affect the structure of ecological networks, including food webs. These ecological network studies partly support our conclusion. In this study, we did not perform such a network analysis because we were not able to construct food-web networks. In particular, the data we used on predator–prey interactions partly consisted of prey species for which descriptions do not exist (i.e. they are expressed as species A and species B) or are ambiguous.

In addition to the stated limitations, our analysis has a primary limitation, shared by many other studies on ecological interaction analyses. For example, knowledge remains limited on interspecific interactions (i.e. missing links). We did not consider the effect of phylogenetic signals, even though a previous study [67] found a significant phylogenetic signal in the analyses of prey body-mass range for predators and predator body-mass ranges for prey, with a stronger signal in the former. As pointed out in a previous study [29], more focused analyses on body size and species identity across food webs in different ocean environments are needed to address this issue.

To overcome these limitations, larger-scale and more highly normalized databases should be constructed. In this context, data sharing [68] will be important. Moreover, sequencing analyses also play an important role, and are now beginning to be applied to ecology (e.g. in population ecology [69,70] and identifying species–species interactions [71]). Sequencing analysis-based approaches, such as DNA barcoding [72] and DNA-based gut content analyses [73], have also been used to detect predator–prey interactions in terrestrial and marine systems, even though several limitations exist.

In conclusion, despite the limitations in our data analyses, the findings of the current study advance our understanding of the effects of ocean environments (climate change, in particular) on the structure and function of marine food webs.

## Supplementary Material

Table S1

## Data accessibility

The datasets supporting this article have been uploaded as the electronic supplementary material.
